# Optimized Conditions for Extracting Native Type-I Collagen from Discarded Fish Skin Using Hydrochloric Acid to Overcome the Drawbacks of Acetic Acid

**DOI:** 10.3390/md24010028

**Published:** 2026-01-08

**Authors:** S.T. Gonapinuwala, J.R. Jones, S. Kirk, M.D.S.T. de Croos, J.E. Bronlund

**Affiliations:** 1 School of Food and Natural Sciences, Massey University, Palmerston North 4442, New Zealand; j.r.jones@massey.ac.nz (J.J.); steve.kirk@medicalcollagennz.com (S.K.); 2 Department of Aquaculture and Fisheries, Faculty of Livestock, Fisheries and Nutrition, Wayamba University of Sri Lanka, Makandura, Gonawila 60170, Sri Lanka; dileepad@wyb.ac.lk

**Keywords:** fish skin, homogenization, hydrochloric acid, pH, swelling, triple helix

## Abstract

Fish skin, a by-product of commercial fish processing, represents a viable source of type I collagen. Acetic acid has been widely used for the extraction of collagen from fish skin because it can preserve the native structure. However, it requires an extraction time of more than 72 h and complex and time-consuming dialysis steps to remove acetic acid residues from the extracted collagen which can otherwise cause inferior structural modifications. Therefore, this study describes a simple time- and cost-effective method to extract collagen using hydrochloric acid. The experiments focused on understanding the behavior of fish skin and changes in the extraction medium. The extraction procedure developed in this study includes treatment with a 0.01 M hydrochloric acid solution at a 1:20 mass to volume ratio for 5 h, followed by homogenization. The native triple-helical structure of collagen was confirmed by ATR-FTIR and circular dichroism spectroscopy. Thermal stability was confirmed by differential scanning calorimetry. This study also provides guidelines for the application of this knowledge to skin of any fish species of interest: (i) an upper limit of pH 4 during collagen extraction; (ii) a manageable viscosity of the collagen extract solution; and (iii) as few undissolved skin pieces as possible after homogenization.

## 1. Introduction

Industrial fish processing discards 50–80% of the raw fish as by-products, causing environmental problems and economic losses [1,2,3,4]. Fish skin is a major component of these by-products, containing more than 70% type I collagen on a dry weight basis [5], which has a wide range of applications in the biomedical industry such as wound dressings, drug delivery systems, gene delivery systems, and tissue engineering systems. Consequently, fish skin presents a viable source of type I collagen. In addition, fish-origin collagen is associated with a lack of known risk of disease transmission and fewer religious barriers [6,7], showing its high potential as an alternative source for mammalian-origin collagen. Furthermore, fish-origin collagen is superior to mammalian-origin collagen in biocompatibility, biodegradability, low antigenicity [8,9,10], high cell growth [11], high cell adhesion and high cell proliferation [12,13]: properties which are essential in biomedical applications. However, this valuable resource has not been exploited to its full potential, mainly due to the unavailability of industrial-scale processing methods that extract collagen while preserving the native triple-helical structure which is required for biomedical applications [14].

Collagen is a right-handed supercoiled triple-helical molecule composed of three left-handed α-helices. The stability of this super-helical structure is maintained through the steric hindrance or restrictions on conformational changes imposed by the pyrrolidine rings of proline and hydroxyproline amino acids [15]. The intramolecular hydrogen bonds formed through the hydroxyl groups of hydroxyproline by a bridging water molecule and direct hydrogen bonding to carboxyl groups also help to stabilize the triple helix [15]. When these collagen molecules are arranged into fibers, lysine-derived covalent cross-links form between the non-helical end of one collagen molecule and the helical end of another collagen molecule [16].

Prior to collagen extraction, fish skin must be pretreated to remove non-collagenous protein, fat and pigments. Collagen extraction begins with the hydration and swelling of the collagen fibers, which facilitates the exposure of collagen to the extraction medium [17]. During swelling, intermolecular covalent cross-links are cleaved and collagen molecules become soluble in the medium, but they should retain their triple-helical structure [18]. It is therefore important to preserve the intramolecular hydrogen bonds during collagen extraction. The dominance of positive charges in collagen molecules under acidic conditions causes increased repulsion between collagen molecules, which leads to increased solubilization [19]. This dissolution step is necessary for these collagen molecules to self-assemble back into fibers during the subsequent fibrillogenesis step. The most important factor is that the solubilized collagen should remain in its native triple-helical conformation [11]. If this structure is broken or the collagen molecules are hydrolyzed, they cannot self-assemble into fibers with the required D-spacing, which is essential for biomedical applications [10].

Many researchers have described the importance of a mild chemical treatment, mainly using acetic acid for collagen extraction. But acetic acid has drawbacks in biomedical collagen extraction. Due to the incomplete ionization nature of acetic acid, it can be adsorbed onto collagen molecules and can remain as residues in the final freeze-dried collagen product [11]. Bak et al. [20] reported that acetic acid residues make larger pores with weaker pore walls in collagen scaffolds during freeze-drying, which causes inferior structural modifications. Therefore, traditional extraction methods using acetic acid—for example, those used by Xu et al. [5], Li et al. [6], Ampitiya et al. [21], and Wang et al. [22]—require dialysis steps to remove the acetic acid residues, which are associated with additional costs in industrial applications. In addition, extraction of collagen with acetic acid generally involves longer extraction times, usually 72 h for extraction and another 72–96 h for dialysis, as shown by Ahmed et al. [15], Ampitiya et al. [21], and Muralidharan et al. [23], which is another drawback for industrial-scale processing.

A low-concentration HCl solution has been tested by fewer researchers as an alternative for acetic acid. According to Skierka and Sadowska [17], a 0.15 M HCl solution (pH 0.87) was the least effective at producing a higher yield compared to acetic acid. This failure can be a reason for high acidity. Unlike acetic acid, HCl ionizes completely to H^+^ and Cl^−^ ions in the solution, whereas only the H^+^ ions adsorb to collagen molecules in low HCl concentrations [24]. But in very high acidic conditions (below pH 2) Cl^−^ ions adsorb to collagen molecules, which can lead to decreased hydration, decreased swelling, decreased solubility and low yield [17]. According to Zhou et al. [24], HCl solutions below pH 2.0 failed to preserve the triple-helical conformation. In the study by Gonapinuwala et al. [25], collagen extracted from tarakihi fish skin using HCl acid at pH 2 confirmed the triple-helical structure; however, an optimization of extraction conditions was not performed. That study focused on determining the optimum pH and the other conditions required for optimum fibrillogenesis. But in another study by Gonapinuwala et al. [26], ling fish skin collagen and yellowfin tuna fish skin collagen extracted using HCl acid at pH 2 preserved the triple-helical structure, and collagen extracted from tarakihi fish skin using the same extraction conditions resulted in only a 7.71% yield on a dry weight basis; further, it failed to preserve the triple-helical structure. Although that study compared the characteristics of collagen extracted from different fish species, no investigations were conducted on optimizing the extraction conditions. Therefore, understanding the optimum conditions required for effectively extracting collagen by using HCl acid from fish skin while preserving the triple-helical structure is a timely requirement.

On the other hand, there is a limited amount of research describing the process of collagen solubility; however, no studies were found describing the behavior of fish skin in the extraction medium. This knowledge is important to design efficient extraction processes using HCl acid. Therefore, this study fills this gap by describing the hydration, swelling and solubility of fish skin in an HCl acid solution. For this, a set of novel experiments were designed to optimize the extraction conditions by analyzing the effect of the extraction time and mass to volume ratio of fish skin in HCl acid solution on the solubility of the skin and pH changes and viscosity in the HCl acid medium. Most importantly, the method of extraction should be capable of extracting the collagen in its native triple-helical structure for biomedical applications [27]. Therefore, a more detailed characterization of collagen, using different standard techniques such as ATR-FTIR, circular dichroism spectroscopy, differential scanning calorimetry, and SEM, was performed in this study to determine the effect of the extraction conditions of HCl acid on the triple-helical structure and its stability, by comparing it with the reference values of native triple-helical collagen. This approach provides advantages over comparing it with collagen extracted using acetic acid, as our main focus was not only to determine the effectiveness of HCl as an alternative to acetic acid, but also to confirm the preservation of the native triple-helical structure in extracted collagen. The time- and cost-effectiveness and the minimum use of chemicals, water and energy were considered when selecting the suitable processing conditions for the developed extraction process. In this study, only the structural characterization required for biomedical collagen was considered, but the biological or functional characterization, such as cytocompatibility, cell adhesion or in vitro bioactivity tests were not considered.

## 2. Results

### 2.1. Suitable pH for Collagen Extraction, Using HCl as the Extraction Medium

**Figure 1:**
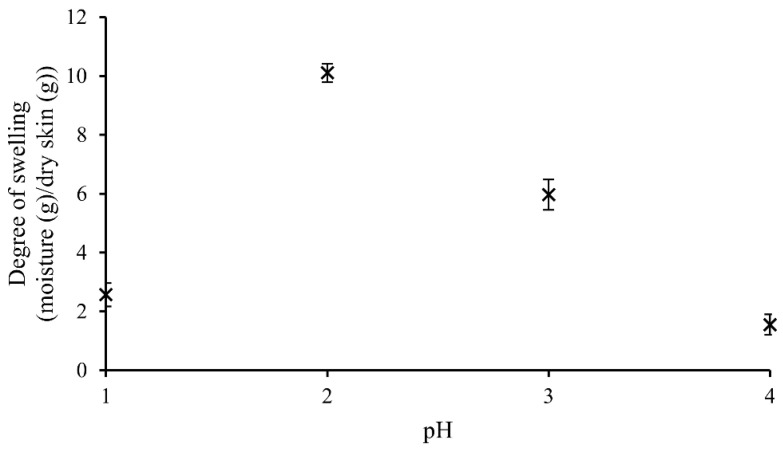
Swelling of fish skin at different pH levels of HCl acid. Means with standard deviations are given.

The degrees of swelling of fish skin at different pH levels ranging from pH 1 to pH 4 are shown in Figure 1. Swelling is required to facilitate the solubilization of collagen molecules by exposing them to the extraction medium. Therefore, pH 2 (0.01 M concentration), which gave the highest swelling, was selected as the suitable pH for collagen extraction.

### 2.2. pH, Solubility and Viscosity During Extraction

#### 2.2.1. pH of the Solutions During Extraction

Table 1 shows the pH values of collagen extracts at different stages of extraction. The theoretical pH of 0.01 M of the HCl solution used as the extraction medium was pH 2. The initial pH values of the HCl solutions and the pH values at three different stages of extraction (by the end of each extraction time, by the end of homogenization and before starting the fibrillogenesis), as measured by the pH meter, are given in Table 1.

Table 1 clearly shows an increase in pH throughout the extraction process, irrespective of the extraction conditions. Further, different extraction conditions have led to different pH levels at different stages of extraction.

#### 2.2.2. Solubility of Fish Skin During Extraction

**Figure 2:**
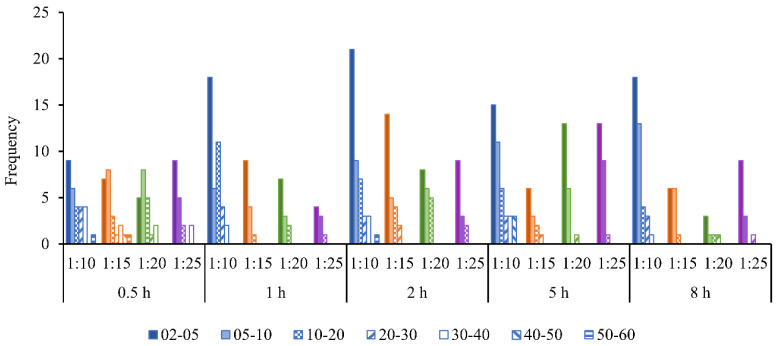
Frequency distribution of undissolved skin pieces after homogenization for all treatments of the combination of extraction time (0.5 to 8 h) and skin mass to solution volume ratio (1:10 to 1:25). Skin surface area categories are 2–5, 5–10, 10–20, 20–30, 30–40, 40–50 and 50–60 (mm^2^). The legend shows the shading for the 1:10 ratio in blue. The other ratios follow the same shading with different colors: orange for 1:15, green for 1:20 and purple for 1:25.

A simple method was introduced in this study to qualitatively measure the solubility of the skin by measuring the number of undissolved skin pieces and their surface areas after homogenization. For the analysis, seven categories of surface area were selected—2–5, 5 –10, 10–20, 20–30, 30–40, 40–50 and 50–60 mm^2^—and the number of skin pieces in each category were counted and presented as a frequency distribution (Figure 2).

Figure 2 shows that there are more small-area skin pieces than large-area ones. In addition, there are three trends: (i) at 0.5 h extraction time, the count distribution is unaffected by the skin mass to solution volume ratio; (ii) at 1 h extraction time, the overall count reduces as the ratio decreases; and (iii) for all results across extraction times from 1 to 8 h, there are many more skin pieces at a skin to solution ratio of 1:10 than at 1:25.

#### 2.2.3. Viscosity of the Solutions During Extraction

**Figure 3:**
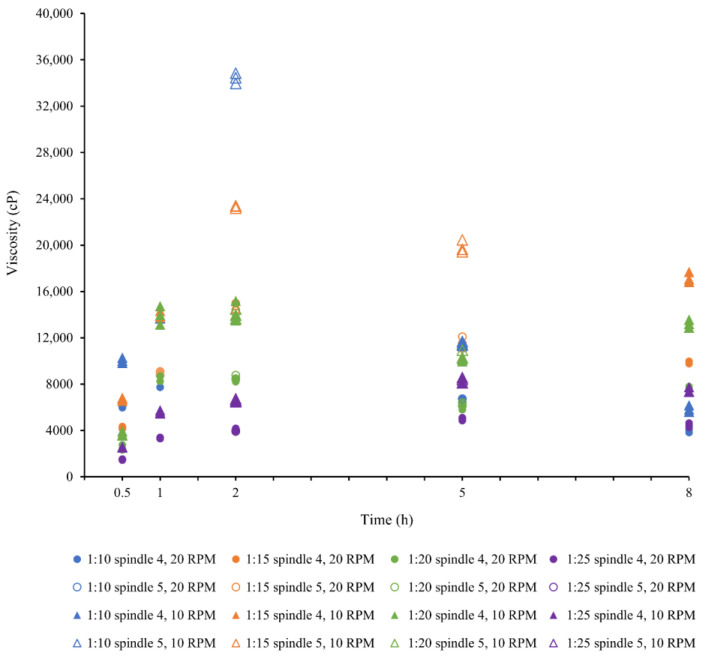
Viscosity of homogenized collagen solutions measured by spindle number 4 at 10 rpm. Spindle number 5 was used to measure the viscosity above 20,000 cP. Note: Direct comparison of Spindles number 4 and 5 are not theoretically the same. So, there will be small differences in shear rates. The 2 h and 5 h treatment times at the 1:20 (*m*/*v*) ratio show they are comparable.

Viscosity of homogenized solutions are shown in Figure 3.

The viscosity of the homogenized collagen solutions was measured by spindle 4 at 10 rpm. Spindle 5 was used to measure viscosities above 20,000 cP. As shown in Figure 3, viscosity values recorded for the 2 h, 1:20 (*m*/*v*) treatment by both spindle numbers 4 and 5 were very similar, as shown by the green triangles. Similarly, viscosity values recorded for the 5 h, 1:20 (*m*/*v*) treatment by both spindles were also very similar. This may be due to the very small differences found in the shear rates between these two spindles because of their very similar diameters. Therefore, it is reasonable to use spindle number 5 to measure viscosities that are not possible with spindle 4.

The results show that the 1:15, 1:20 and 1:25 ratios have progressively lower viscosity at all extraction times because the dissolved collagen is more dilute in the solution. However, of great interest is the peak in viscosity, which occurs at 2 h extraction time, and decreases thereafter.

### 2.3. Morphology and Microstructure of Collagen

**Figure 4:**
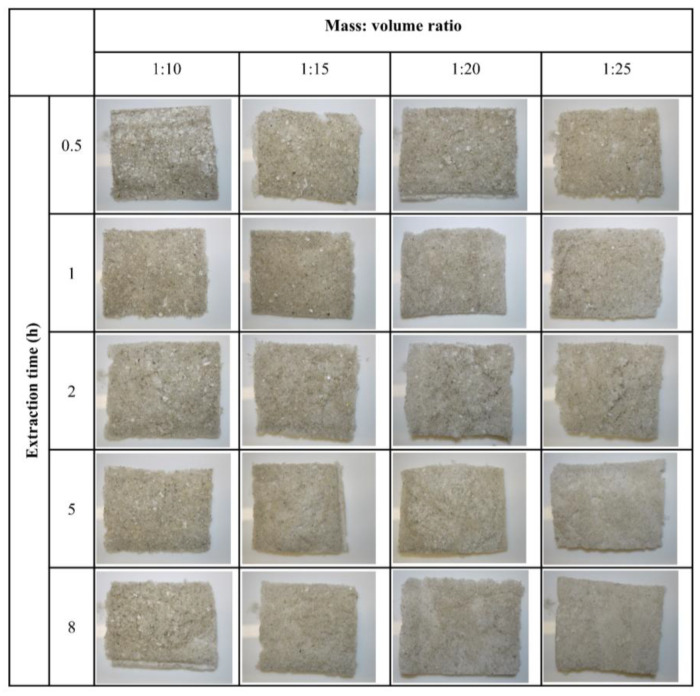
The optical photographs of freeze-dried collagen after fibrillogensis, with respect to extraction time, and the fish skin mass to solution volume ratio.

Optical photographs of the collagen are shown in Figure 4. Undissolved skin pieces are clearly seen in the top row for the 1:10 ratio solution, but a few are absent in the 1:20 and 1:25 ratios at the 5 and 8 h extraction times.

**Figure 5:**
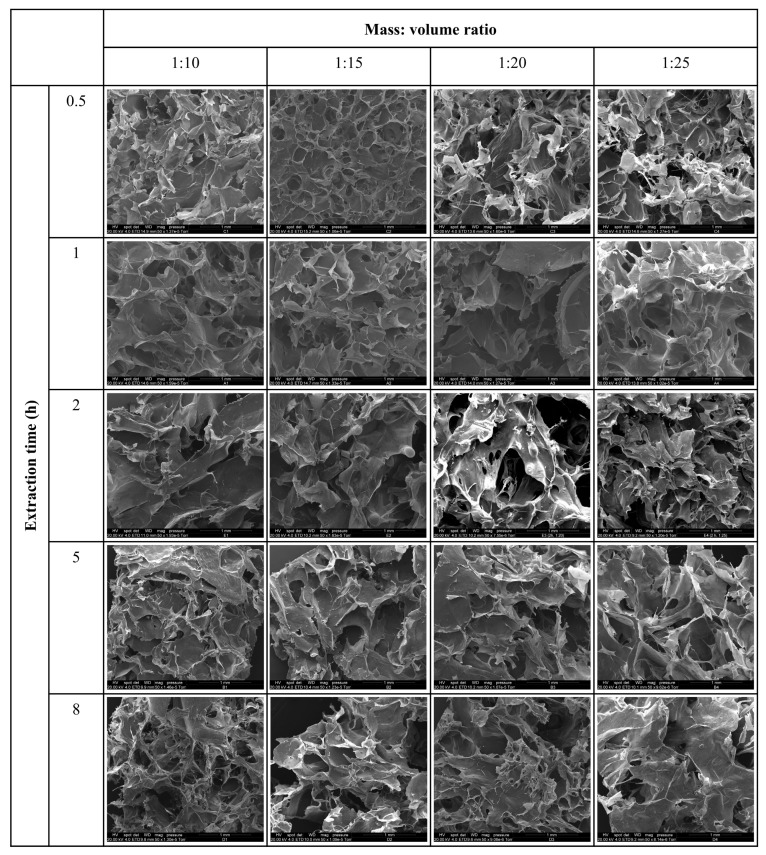
SEM images (×50) of freeze-dried collagen after fibrillogensis, with respect to extraction time, and the fish skin mass to solution volume ratio.

The surface microstructures of freeze-dried collagens were visualized using scanning electron microscopic (SEM) images at a low magnification (Figure 5). All images show a sheet-like layering with a porous structure produced as a result of the sublimation of water during freeze-drying. Martins et al. [28,29] also reported porous structures in collagen produced as a result of freeze-drying.

### 2.4. Native Triple-Helical Structure of Collagen

The secondary structure of collagen was analyzed by the attenuated total reflectance–Fourier transform infra-red (ATR-FTIR) spectroscopy and circular dichroism (CD) spectroscopy techniques.

#### 2.4.1. ATR-FTIR Spectra of Collagen

**Figure 6:**
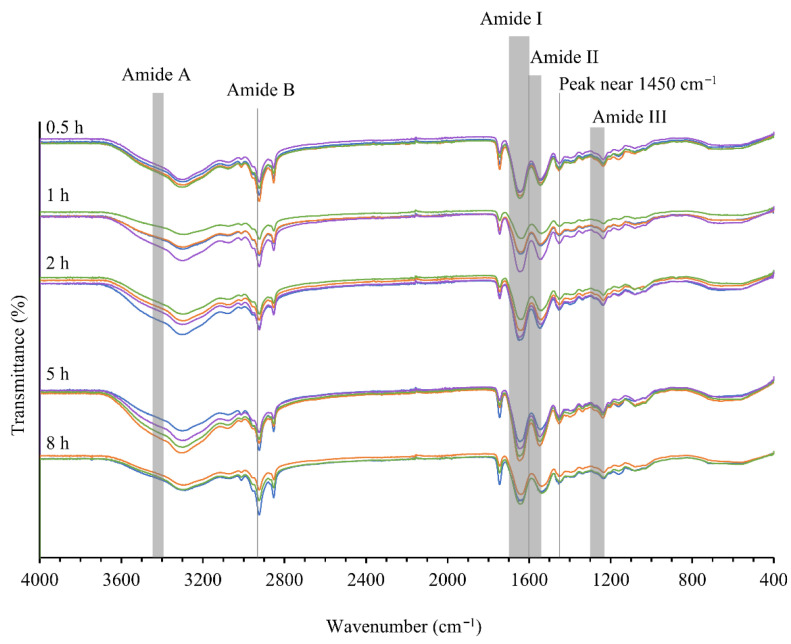
ATR-FTIR spectra of freeze-dried collagen extracted at five extraction times and four mass to volume ratios. Graphs represent the mean of five replicates. Blue represents 1:10, orange represents 1:15, green represents 1:20 and purple represents 1:25.

ATR-FTIR is used to determine the type of collagen [8] and the changes in the secondary structure of collagen, based on the chemical bonding state [22,30,31]. The peak locations of all 20 collagen samples are given in Figure 6 and Table 2. Amide A, B, I, II, and III peaks were present for all samples, which is typical of Type I collagen. However, the peak positions showed slight differences, indicating some differences in the secondary structure [22].

#### 2.4.2. CD Spectra of Collagen

**Figure 7:**
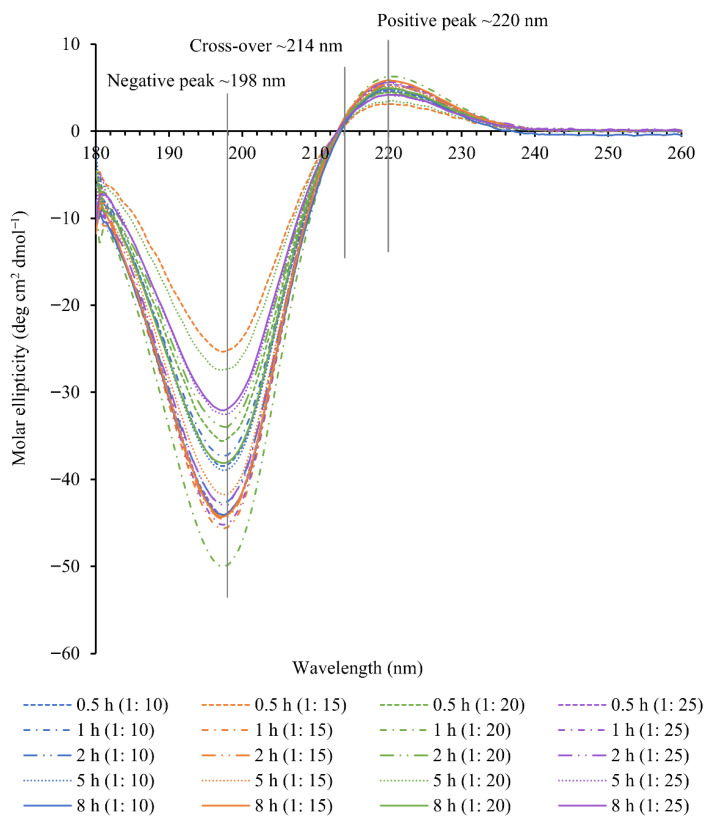
Circular dichroism spectra of collagens.

The CD spectra obtained for twenty different treatment combinations are shown in Figure 7. The secondary structure determination by this technique is based on the measurement of differential absorption of left- and right-handed polarized light by peptide bond chromophores in a protein [11,24,32] in the far UV range, generally from 250 to 190 nm [33]. Circular dichroism spectroscopy is considered the gold standard for estimating the secondary structure of proteins among other techniques, due to the easy identification of the native and denatured conformations [34]. The easy identification of the native structure from the denatured structure is based on the presence or absence of the positive peak as it disappears upon denaturation [32]. The native triple-helical structure of collagen in a solution is characterized by the presence of a negative peak at around 198 nm related to the content of the α-helix structure, a cross-over at 214 nm and a weak positive peak at around 220 nm due to the π-π* transition of peptide bonds. All collagens have produced characteristic CD spectra with a negative peak at ~198 nm, a cross-over point at ~214 nm and a positive peak at ~220 nm. The same values have been reported in previous studies: for example, Zhou et al. [24], Gonapinuwala et al. [25], and Liu et al. [35].

The calculated value of the ‘positive/negative ratio’ (Rpn) from the CD spectra is used as a measure to estimate the degree of denaturation or the triple-helical content [36,37]. The Rpn ratios of 20 samples are shown in Table 3. If the collagen is denatured, the Rpn ratio is a positive value and it increases with an increasing degree of denaturation [37].

### 2.5. Thermal Stability of Collagen

**Figure 8:**
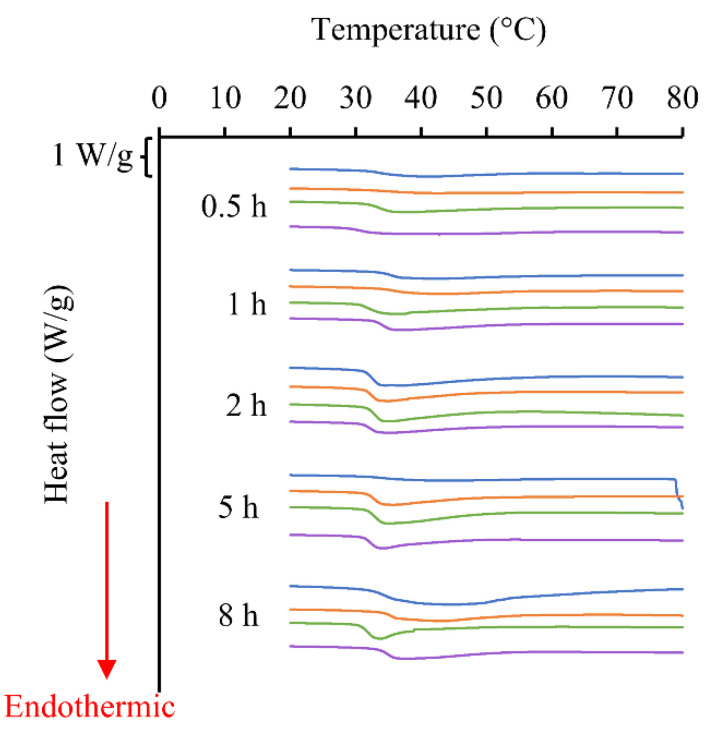
Differential scanning calorimetry thermograms of collagens. Blue represents 1:10, orange represents 1:15, green represents 1: 20 and purple represents 1:25.

The denaturation temperature (Td), onset temperature and denaturation enthalpy (∆H) of collagen samples measured using the differential scanning calorimetry (DSC) thermograms are given in Table 3 and Figure 8. The 1:10 and 1:15 mass to volume ratios have higher Td values. Other ratios have values of around 34–38 °C.

### 2.6. Yield of Collagen

The yield of collagen for all extraction conditions was between 68 and 74% on a dry weight basis.

## 3. Discussion

### 3.1. pH, Solubility and Viscosity During Extraction

In most of the literature, the initial pH or the concentration of the chosen acid has been mentioned; however, the changes in pH during extraction have not been discussed with respect to its effect on collagen dispersion. This is addressed here. The collagen extraction process starts by swelling the skin, thus facilitating the exposure of collagen fibers to the extraction medium. This allows the acid to promote the dispersion of collagen fibers to colloidal particles by breaking the intermolecular covalent cross-links between collagen molecules. Complete ionization of HCl provides H^+^ and Cl^−^ into the extraction medium. Some of these H^+^ ions adsorb onto the alkaline side chain radicals (-NH2) of collagen molecules and the rest are free in the solution. This adsorption of H^+^ decreases the concentration of H^+^ in the solution, therefore increasing the solution’s pH. The H^+^ adsorption also increases the net positive charge, which increases the electrostatic repulsion between different parts of the collagen colloidal particles [6,11,19,38]. This repulsion drives further breakdown into even smaller particles and into collagen molecules. This exposes more alkaline side chains and consequently more H^+^ adsorption, thus further increasing the solution’s pH [11].

There was a time gap between the end of homogenization and the start of fibrillogenesis, due to the time required to perform the viscosity measurements, as reported later, and so the pH was recorded again because further dissolving of collagen molecules into the solution had occurred. As the pH approaches the isoelectric point of collagen, it decreases the net positive charges and so decreases the repulsive forces between molecules, thus decreasing the solubility and forming collagen aggregates [38,39]. Therefore, pH conditions close to the isoelectric pH of collagen are not suitable for collagen extraction. Previous studies have reported less solubility of collagen above pH 4, due to the approach to the isoelectric pH of collagen. For example, Song et al. [40] reported a remarkable decrease in the solubility of Nile tilapia skin collagen from pH 4 to 7; and Rodríguez et al. [38] reported maximum solubility between pH 2–4 and a decrease in solubility between pH 4–8 for mussel byssus collagen. In the experiments reported in Table 1 and marked in red, the 1:10 mass to volume ratio for all five extraction times and the 1:15 mass to volume ratio for both 5 and 8 h extraction times exceeded pH 4 during extraction. This will be discussed alongside the other results later.

As noted earlier, although solubility increases with increasing acidity, this also has a limit. Very high acidic conditions are not suitable due to their denaturing effect, as reported by Qi et al. [11] for pH 3 and Ahmed et al. [15] for pH 2. In these experiments, the initial pH was 2, which is on the border of causing denaturation, according to these authors; however, here, the pH quickly increases due to the removal of the H^+^ ions from the solution as alkaline side chain radicals become exposed. Therefore, it can be assumed that it is the pH during extraction that is important in deciding extraction conditions, rather than the initial pH. According to the results on preservation of the native triple-helical structure of collagens, which will be discussed later, all collagens, extracted by twenty different extraction conditions, were found in their native form without denaturation. Nevertheless, it is possible that there is an initial pH that is too acidic and initiates denaturation rather than adsorption. If so, this work shows that it is not pH 2 but must be some lower but undetermined value.

Because the pH has a relationship with the solubility of the collagen, it is important to discuss them together. As described above, previous studies have reported the effect of pH on the solubility of collagen; however, no studies were found describing the effect of pH on collagen dispersion from fish skin. Therefore, as shown in the results section, a simple method was introduced in this study to qualitatively measure the solubility of the skin. The results can be anticipated from the expected chemistry. More skin should dissolve in the extraction medium with increasing extraction time (from 0.5 to 8 h) and with increasing solution (from 1:10 to 1:25 skin mass to solution volume ratio). Homogenization then mechanically breaks down the remaining undissolved skin pieces, reducing large skin pieces to form smaller pieces. The particle area analysis indicates the efficacy of dissolution and homogenization in reducing the skin piece size.

The results in Figure 2 approximately follow this expectation, where there are more small-area skin pieces than large-area ones. In addition, there are three trends: (i) at 0.5 h extraction time, the count distribution is unaffected by the skin mass to solution volume ratio, which suggests insufficient time for the solubilization of skin pieces; (ii) at 1 h extraction time, the overall count reduces as the ratio decreases, which suggests that there is sufficient time for solubilization so that the effect of the amount of solution becomes important; and (iii) for all results across extraction times from 1 to 8 h, there are many more skin pieces at a skin to solution ratio of 1:10 than at 1:25, which suggests that the 1:10 ratio provides insufficient hydrogen ions for the required adsorption, and which is also supported by the finding that the pH of these solutions rises above 4 (Table 1), indicating depletion in the availability of H^+^ ions. Interestingly, trend (ii) does not carry through to the longer extraction times of 2, 5 and 8 h, but this could be statistical, as the total number of measured skin pieces was not high.

These solubility results can be further interpreted with respect to the viscosities of the homogenized solutions, which are shown in Figure 3. Viscosity affects two aspects—the molecular mobility and the ease of mixing—which are both important in the next step, fibrillogenesis, where the collagen fibers are formed from the acid solution by the addition of NaOH solution to raise it to close to its isoelectric point. When the viscosity of the solutions is too high, it reduces both the ease of mixing, and therefore mixture uniformity, during the fibrillogenesis, and the molecular mobility for self-assembly [10,41,42].

Solution viscosity is also expected to affect the swelling of the skin and the solubilization of collagen molecules. Swelling of the skin occurs by absorbing the solution, which is easier when the solution has low viscosity. As described before, swelling enables breaking down the inter-molecular covalent cross-links and dispersion of collagen molecules into the extraction medium. Low viscosity will provide good molecular mobility for diffusion of the H^+^ ions to the adsorption sites and the movement of the dissolved collagen molecules back into the solution. However, the gradual increase in dissolved collagen content in the solution is expected to increase the solution viscosity over time. This is supported by the results, which show that the 1:15, 1:20 and 1:25 ratios have progressively lower viscosity at all extraction times because the dissolved collagen is more dilute in the solution. However, of great interest is the peak in viscosity, which occurs at the 2 h extraction time, and thereafter decreases, which suggests that even in the solution, the collagen is undergoing further cleavage of cross-links. The 1:10 ratio, as noted before, appears to not have enough H^+^ ions for the adsorption phase of the dissolution, and so the development of viscosity is limited.

These results provide important information about the influence of the process conditions on the efficacy of collagen extraction, with a particular focus on the solution. The processing objectives are to have the following: (i) an upper limit of pH 4 for the extraction solution; (ii) a manageable viscosity of the extraction solution; and (iii) as few undissolved pieces of skin as possible. Considering all these factors, and the cost- and time-effectiveness, the 5 h extraction time at a 1:20 mass to volume ratio was provisionally selected as the optimal extraction condition. The next section moves from analyzing measurements of the solution to measurements of the produced collagen.

### 3.2. Morphology and Microstructure of Collagen

Undissolved skin pieces seen in the optical photographs align with the counting of the undissolved skin pieces in the solution after homogenization, as shown in Figure 2. The freeze-drying conditions were kept constant for all samples, which means differences in pore structure are likely to reflect differences in the collagen networks. However, no differences can be seen in these images to conclude any effect from the extraction time or mass to volume ratio.

### 3.3. Native Triple-Helical Structure of Collagen

Amide A, B, I, II, and III peaks were present for all 20 collagen samples, which is typical of Type I collagen, although the peak positions showed slight differences, indicating some differences in the secondary structure [22].

The amide A peak is due to the stretching vibration of the N-H bond in the 3400–3440 cm^−1^ region [6,22,31]. This peak shifts to a lower wavenumber when the N-H group is involved in hydrogen bonding with a carbonyl group of a peptide bond from another polypeptide chain [8,25]. All collagens have shown decreased wavenumbers towards 3300 cm^−1^, indicating more hydrogen bonds, which is a good indication of the triple-helical collagen structure. All collagens have shown a strong peak around 2925 cm^−1^ due to the asymmetrical stretching of CH2 bonds, which is responsible for amide B [26,43].

The amide I peak is found in the 1600–1700 cm^−1^ region, due to the C=O stretching vibration [44]. The formation of H bonds between N-H (X position) and C=O (Gly) of the fourth residue is responsible for the triple-helical conformation and is shown by the shifting of the peak position to a lower frequency [6,25]. The amide I peak position is the most useful measure, which allows for a better differentiation of the secondary structures [39] (: 1620–1640 cm^−1^ (β sheet or extended structure); 1640–1644 cm^−1^ (irregular structure); 1645–1659 cm^−1^ (α-helix); and 1660–1700 cm^−1^ (β turn) [45]. The wavenumbers for all twenty collagens were found in the 1645–1659 cm^−1^ region, suggesting the α-helix structure. There are some peaks found that are related to the β sheet and irregular structures, but this is negligible compared to the content of the α-helix structure.

The N-H in-plane bending coupled with C-N stretching vibrations is responsible for the amide II peak in the 1550–1600 cm^−1^ region [31]. Here, the peak position shifts to a lower wavenumber if more hydrogen bonds are present between adjacent α-chains [6]. All collagen samples have recorded peak positions around or below 1550 cm^−1^, indicating more hydrogen bonds.

There is another implication for the shift in wavenumber for the amide I and II peaks, which is that of cross-linking, where higher wavenumbers indicate a higher degree of intermolecular cross-links and molecular order [6]. This means the measured wavenumbers are subject to two opposing influences. Here, the net result is that hydrogen bonding dominates and so has decreased the wavenumbers. The consequence of more hydrogen bonding is that peptide chains are less likely to unwind [6] and so preserve the secondary structure of proteins.

The amide III band occurs in the 1230–1300 cm^−1^ region, mainly due to N-H bending coupled with C-N stretching and wagging vibrations from CH2 groups of the glycine backbone and proline side-chains [5,6,15,31]. All collagens have recorded peaks in the 1230–1238 cm^−1^ region. Another strong peak occurs near 1450 cm^−1^ due to the pyrrolidine ring vibrations of proline and hydroxyproline [44]. A useful parameter which is called the absorption ratio is calculated from the ratio of the amide III and 1450 cm^−1^ absorptions to determine the intactness of the triple-helical structure [6,9]. This ratio is close to 1 for the triple-helical structure [5,40,43] and close to 0.5 if the structure is denatured [26,46]. The absorption ratios calculated for twenty samples are shown in Table 3, which are around 0.85, confirming the triple-helical structure [47]. These results confirm that the extraction conditions used in this study were able to maintain the native triple-helical conformation of collagen up to 8 h.

All 20 collagen samples have produced characteristic CD spectra of native collagen with a negative peak at ~198 nm, a cross-over point at ~214 nm and a positive peak at ~220 nm. The calculated value of the ‘positive/negative ratio’ (Rpn) from the CD spectra, which is used as a measure to estimate the degree of denaturation or the triple-helical content, were found in the range from −0.11 to −0.13 (Table 3). If the collagen is denatured, the Rpn ratio is a positive value and it increases with an increasing degree of denaturation [37]. For example, Gopinath et al. [34] and Gonapinuwala et al. [26] reported Rpn ratios of 0.05 and −0.03, respectively, for denatured collagen. The Rpn ratios reported in previous studies for native collagens were −0.13, −0.12, −0.11, −0.1075 and −0.123, by Gonapinuwala et al. [26], Gopinath et al. [34], Gonapinuwala et al. [26], Nishad Fathima et al. [48] and Sun et al. [27], respectively. The values reported in this study fall within the values of previous studies reported for native collagen. Therefore, these results indicate that the extraction durations and mass to volume ratios used in this study were suitable for preserving the triple-helical structure.

### 3.4. Thermal Stability of Collagen

The thermal stability of the triple-helical structure of collagens is stabilized by the intramolecular hydrogen bonding between the hydroxyl group of hydroxyproline in one polypeptide chain and the main chain amide carboxyl group of another chain, mediated by water molecules [15,19,49]. The thermal denaturation process of collagen starts by first breaking these intramolecular bonds upon heating, which unravels the tightly bound triple helix into looser polypeptide chains, followed by disintegrating it into random coils at a particular temperature, which is defined as the denaturation temperature [35,50]. This thermal denaturation results in irreversible damage in the physico-chemical and biological properties of collagen.

These thermal properties are measured in DSC by a direct measurement of heat [51]. There are two characteristic temperatures measured in DSC: the denaturation temperature, which corresponds to the temperature of maximum power absorption during denaturation, providing a sharp peak in the thermogram, and the onset temperature, which is the temperature at which the tangent to the initial power versus temperature line crosses the baseline [52]. In addition, the denaturation enthalpy, which is calculated from the area under the peak corresponding to the total heat involved in the transition, is also used [52].

The denaturation temperature (Td), onset temperature and denaturation enthalpy (∆H) of collagen samples are given in Table 3 and Figure 8. The 1:10 and 1:15 mass to volume ratios have higher Td values, which may be due to the presence of undissolved skin pieces, which is not acceptable. Other ratios have values of around 34–38 °C. Kittiphattanabawon et al. [53] and Nalinanon et al. [54] reported Td values of around 30 °C and Gonapinuwala et al. [26] reported a Td around 43 °C for warm-water fish collagen. However, the Td of cold-water fish collagen is mostly below 20 °C [6,53]. Gonapinuwala et al. [25,26] reported a Td around 41 °C and 43 °C for warm-water fish collagen. Accordingly, this study reported higher Td values for collagen extracted from cold-water fish skin.

There are several factors affecting the DSC results which should be considered in the sample preparation and choice of method, such as the pH and concentration of the medium, heating mode [50], the water content in the sample, and the presence of admixtures [51]. Considering these factors, it can be argued that the higher Td values reported in this study were not due to any of these, as the source of collagen, sample preparation and analysis method were the same.

The onset temperatures were comparatively lower and varied for extraction durations up to 2 h, while much more consistent and higher values (25–30 °C) were observed for 5 and 8 h. This may be due to the presence of more homogenous solutions consisting of collagen molecules after 5 and 8 h extractions compared to the mix of skin and collagen molecules in the extracts up to 2 h, as explained before. These results imply the possibility of using processing temperatures of up to 25 °C without any thermal degradation effect on collagen. This is important in practical handling and further processing into biomedical materials, since the physico-chemical, mechanical and biological properties are destroyed by thermal denaturation [55].

There was no relationship found between Td and denaturation enthalpy. Previous studies have reported that a higher denaturation enthalpy is associated with a higher content of the Gly-Pro-Hyp sequence in the polypeptide chains [15,56]. This is due to the higher stability of hydrogen bonds between the polypeptide chains of this Gly-Pro-Hyp sequence compared to other sequences [15,57]. However, the different denaturation enthalpy values reported in this study cannot support this argument; hence, further studies are needed.

### 3.5. Yield of Collagen

The yield of collagen obtained from the selected extraction conditions of 5 h at a 1:20 mass to volume ratio was 69.7 (±0.5)% on a dry weight basis, which is a promising factor for upgrading to commercial-scale processing. Yield was also recorded for all other extraction conditions and was between 68 and 74% on a dry weight basis. However, as discussed earlier, some of the extraction conditions were unable to dissolve all skin pieces in the extraction medium, which resulted in them being present in the extracted collagen. Therefore, it is not reasonable to compare yield data between all extraction conditions.

Considering all of the above results, and the time- and cost-effectiveness, selection of the extraction time of 5 h at 1:20 mass to volume ratio is still valid as the best treatment combination to extract collagen in native triple-helical conformation.

## 4. Methods

### 4.1. Sample Preparation

Tarakihi (*Nemadactylus macropterus*), which is a cold-water fish with a high commercial value in New Zealand, was selected for this study. Tarakihi fish skins were collected from a local fish store in Palmerston North, New Zealand as soon the fish were processed and transported to the laboratory in chilled conditions to ensure the freshness and to avoid contamination with extraneous matter. Skins were then cleaned by removing both the scales from the outer surface and the residual flesh left on the inner surface of the skin. Then, the cleaned skins were minced using a meat mincer (Kenwood Pro 2000 EXCEL MG700 Electric Mincer and Sausage Maker, Kenwood Limited, Havant, UK) and washed thoroughly using cold water until the waste wash water ran clean. To ensure uniformity, a random sampling method was used to take 50 g samples from the bulk mince, and they were then pretreated using NaOH to remove non-collagenous proteins and fat, using the method of Gonapinuwala et al. [25]. The weights of the pretreated skins were recorded. After that, they were packed in polyethylene bags and stored at −20 °C until use.

### 4.2. Determination of the Suitable HCl Concentration (pH) for Collagen Extraction

HCl solutions of 500 mL at pH 1 (0.1 M concentration), pH 2 (0.01 M concentration), pH 3 (0.001 M concentration) and pH 4 (0.0001 M concentration) were prepared in triplicates. For each treatment, 50 g of fish skin samples, prepared according to Section 2.1, were soaked in HCl solutions in 2.5 L containers and constantly stirred at 350 rpm using a magnetic stirrer (40 mm long stirring bar). After 1 h, swollen skins were separated from the solution and drained on a 2 × 2 mm strainer for 10 min, and the weight of swollen skins were recorded. This was performed in a cold room at 4 °C.

The degree of swelling for each sample was calculated according to the following equation, where M denotes the mass.

$$Degree\;of\;swelling=\left(\frac{M_{pretreated\;fish\;skin\;at\;the\;end\;of\;soaking\;\left(g\right)-M_{pretreated\;fish\;skin\;at\;the\;beginning\;of\;soaking\;\left(g\right)}}}{M_{Dry\;matter\;in\;pretreated\;fish\;skin\left(g\right)}}\right)$$

The pH level that gave the highest degree of swelling was selected as the suitable pH for extraction in the next experiments.

### 4.3. Experimental Protocol to Select the Suitable Extraction Time and Mass to Volume Ratio

The extraction time in HCl solution and the mass to volume ratio of skin to HCl solution were selected as the independent processing variables. There were 20 treatments used in the experiment by selecting the combinations of five extraction times (0.5 h, 1 h, 2 h, 5 h and 8 h) and four mass to volume ratios (1:10, 1:15, 1:20 and 1:25).

The mass to volume ratio was based on the 50 g of raw skin mass obtained according to Section 2.1. HCl solutions of selected pH from Section 2.2 were prepared separately for each of the 20 treatments. The initial pH of these HCl solutions were recorded. For each treatment, pretreated fish skin samples were soaked in HCl solutions in 2.5 L containers and constantly stirred at 350 rpm, using a magnetic stirrer (40 mm long stirring bar). After the particular extraction time, the pH values of the solutions were recorded.

Then, the swollen skins together with the solution were homogenized, using a hand blender (Kenwood Triblade Systempro, 1000 W, Kenwood Limited, Havant, UK) at a high speed for 15 s, followed by a 60 s rest interval to avoid overheating, repeated 10 times. Homogenization was introduced in this study to first break down the swollen skin into small pieces and to speed up the collagen solubilization because longer extraction times are not cost- and time-effective. Homogenization is a high-shear activity that causes heating and it can cause the denaturation of collagen. Therefore, an undue temperature increase in the extraction medium was avoided by using pulse homogenization. A preliminary experiment was performed, which showed that 10 × 15 s homogenization pulses were able to keep the solution temperature below 10 °C. Nevertheless, incomplete solubilization can leave skin pieces that are visible to the naked eye. This was used as a measure of the homogenization–solubilization efficiency. At the completion of homogenization, a 1.5 mL vial of the homogenized solution was taken from the top middle region to analyze the solubility of the skin as described below in Section 2.5. The homogenization and solubilization produce a viscous solution. The pH values of the homogenized solutions were measured, after which the solutions’ viscosity was measured, using the procedure described below in Section 4.5, and the pH was recorded again, just before fibrillogenesis. For all the samples, the homogenization and viscosity measurements were completed within 40 min.

Collagen fibers were then formed in each homogenized solution by slowly adding a NaOH solution at a 0.01 M concentration. These fibers were then separated and packed in polythene bags, frozen at −20 °C and freeze-dried at 0.5 mbar, 10 °C. The freeze-dried collagen was characterized for morphology, native triple-helical structure and stability. All the experiments were performed in a cold room at 4 °C to avoid the denaturation of collagen by the effect of high processing temperatures.

### 4.4. pH of the Solutions

The pH values of the solutions were measured using a portable pH meter (Thermoscientific, Orion Star A326 pH/RDO/DO meter). It was calibrated according to the manual every time (Thermo Scientific, Waltham, MA, USA, 2015).

### 4.5. Solubility of the Fish Skin After Homogenization

The 1.5 mL vial sample taken from the top middle region of the homogenized solution was put into a glass Petri dish of 14.32 mm diameter. A 7.5 mL volume of distilled water and 1.0 mL of Coomassie blue staining solution were added to the same Petri dish and mixed well using a glass rod. The Petri dish was put in a flat-bed scanner with a dark background and scanned at a 2400 dpi resolution and saved as a color*.jpg image. These images clearly differentiate the undissolved skin pieces from the dissolved skin in the solution. The estimation of the homogenization efficiency was based on the amount of undissolved skin pieces in the 1.5 mL sample. The areas of these undissolved skin pieces were measured using the ImageJ software version 1.53t. The areas that were similar to or higher than 2 mm^2^ were taken as undissolved skin pieces. For the analysis, seven categories of surface area were selected—2–5, 5–10, 10–20, 20–30, 30–40, 40–50 and 50–60 mm^2^,and the number of skin pieces in each category were counted using the ImageJ software. These size categories were compared between different treatments (combination of extraction times and skin mass to solution volume ratios).

### 4.6. Viscosity of the Solutions After Homogenization

The well-mixed homogenized collagen sample was poured to the 500 mL mark of a 600 mL glass beaker and the viscosity was measured using spindle number 4 at 10 rpm in a Brookfield viscometer (Brookfield viscometer DV2T extra, Brookfield Engineering Laboratories, Middleboro, MA, USA). Spindle 5 was used to measure the viscosity above 20,000 cP. Ideally, the temperature was maintained between 9 and 10 °C. The viscosity was measured three times.

### 4.7. Morphology and Microstructure of Collagen

Optical photographs were taken to show the visual differences in collagen under the naked eye. The microstructure of collagens was analyzed by scanning electron spectroscopy. For the SEM, freeze-dried collagen samples were mounted onto aluminum stubs, using double-sided tape to ensure high electrical conductivity between the specimen and the stub. Then, it was sputter-coated with approximately 100 nm of gold (Bal-tec SCD 050 sputter coater, BAL-TEC Inc., Balzers, Liechtenstein) and viewed in the FEI Quanta 200 Environmental Scanning Electron Microscope (FEI Company, Hillsboro, OR, USA) at an accelerating voltage of 20 kV.

### 4.8. Native Triple-Helical Structure of Collagen

#### 4.8.1. ATR-FTIR Spectroscopy

The ATR-FTIR spectra were used to assess the native triple-helical conformation of collagen. The ATR-FTIR spectra were obtained using a Nicolet iS5FTIR spectrometer (Thermo Fisher Scientific Inc., Madison, WI, USA) equipped with an iD7 ATR diamond sampler accessory. A small piece of freeze-dried collagen was placed on the single reflection crystal cell. The signals were automatically collected for 32 scans over the 4000–400 cm^−1^ range, using a 2 cm^−1^ resolution. Background spectra were collected from the clean empty crystal cell. The peak positions of the spectra were analyzed for the triple-helical conformation. Further, the ‘absorption ratio’ was calculated from the ratio of the amide III and 1450 cm^−1^ absorptions to determine the intactness of the triple-helical structure [6,9,22].

#### 4.8.2. CD Spectroscopy

CD spectroscopy was used to assess the native triple-helical conformation of collagen. The freeze-dried collagen samples were dissolved in 0.05 M acetic acid to a concentration of 0.1 mg/mL. Solutions were filtered through a 0.2 µm Millipore filter to remove any undissolved material and dust particles. Then, the mixtures were equilibrated at 4 °C for 48 h before testing. The same concentration of all the samples was verified by measuring the absorption at 192 nm, using a UV-visible spectrophotometer (Genesys 10S spectrophotometer, GENESYS 10S UV-VIS, Thermo Fisher Scientific; Madison, WI, USA) [32]. The degree of ellipticity was recorded with a spectropolarimeter (Chirascan, Applied Photophysics, Leatherhead, UK), using a standard quartz cuvette of 1 mm pathlength (Hellma high-precision cells, Mullheim, Germany). Samples were scanned over a wavelength range from 260 to 180 nm at 0.5 nm intervals. Each sample was scanned five times at a scan rate of 40 nm/min, 0.5 s response time and 1 nm bandwidth. Acetic acid at 0.05 M concentration was used to record the baseline. The same cuvette was used for the baseline and samples. The temperature of the cell holder was maintained at 10 °C, using a temperature control unit (Quantum Northwest). The dynode voltage of all samples was checked to ensure they were below 600 V [32]. Ellipticity measurements for replicates were averaged and the spectrum of the 0.05 M acetic acid solution was subtracted from each sample spectrum. The degrees of the ellipticity values were converted to mean residue ellipticity (deg cm^2^ dmol^−1^) [58] and were graphically presented as a function of the wavelength.

$$\left[\theta \right]=\frac{\theta \left(MRW\right)}{10cd}$$

where $\left[\theta \right]$ is the mean residue ellipticity, θ is the degree of ellipticity (degrees), MRW is the average residue molecular weight, c is the concentration of the sample (mg/mL), and d is the optical pathlength (cm). MRW was taken as 100, assuming the molecular weight of one collagen molecule is about 300,000 and the number of residues/molecule is 3000 [36].

The ‘positive to negative peak ratio’ ($R_{pn}$) was calculated [37].

$$R_{pn}=\frac{\theta_{p}}{\theta_{n}}$$

where $\theta_{p}$ and $\theta_{n}$ are the molar ellipticity values of positive and negative peaks, respectively.

#### 4.8.3. Thermal Stability by DSC

DSC was used to determine the thermal properties of collagen. Small pieces of freeze-dried collagen between 3 and 4 mg were cut and accurately weighed. They were hydrated in a beaker by adding excess cold deionized distilled water. The samples were held underwater for 2–3 min with a clean spatula to speed up the hydration. The hydrated collagen was removed from the water and stuck to the side of the beaker, where they were quickly blotted with filter paper to remove the excess water until it no longer dripped. The hydrated samples were placed into aluminum pans and accurately weighed and hermetically sealed. DSC was performed using a TA Systems Q2000 DSC (TA Instruments, Inc., New Castle, DE, USA). Temperature and enthalpy calibration was performed, using water and indium as standards. The samples were scanned in a nitrogen atmosphere from an initial temperature of 5 °C, held isothermally at this temperature for 1 min. Two identical ramp profiles were subsequentially carried out, consisting of a ramp up at 5 °C/min to 85 °C, then ramp down at 20 °C/min to 5 °C, then held isothermally at this temperature for 1 min, after which the cycle was repeated. Iced water was used as the cooling medium (TA systems refrigerated cooling system 40), and an empty sealed pan was used as the reference. The denaturation temperature was determined from the maximum transition point (the endothermic peak) of the thermal denaturation curve. The total denaturation enthalpy was estimated by measuring the peak area in the DSC thermogram, based on the dry weight of the sample, and expressing this as J/g sample. The onset temperature was also determined, which is the temperature at which the tangent to the initial power versus temperature line crosses the baseline.

#### 4.8.4. Yield of Collagen

The best combination of time and mass to volume ratio was selected from the results of the above experiments and characterization techniques. The yield of collagen obtained using these selected extraction conditions was estimated in triplicates. For this, the dry matter content of the 50 g of raw skin was estimated, using oven-drying at 105 °C for 24 h [59]. For collagen, the reference freeze-dried weight was taken as the dry weight. Yield was calculated as dry collagen (g) per dry weight of raw fish skin (g), according to the following equation, where *M* denotes the mass.

$$Yield=\left(\frac{M_{Freeze-dried\;collagen}}{M_{Dry\;matter\;in\;fish\;skin}}\right)\times 100\%$$

## 5. Conclusions

This study describes the effective processing conditions for the extraction of fish skin collagen in its native triple-helical conformation, using hydrochloric acid as the extraction medium, with particular emphasis on the behavior of fish skin and changes in the extraction medium. The extraction steps developed in this study for collagen extraction from tarakihi skin include extraction with a 0.01 M hydrochloric acid solution at a 1:20 mass to volume ratio for 5 h, followed by homogenization. This study also provides guidelines for the application of this knowledge to the skin of any fish species of interest by providing three processing objectives: (i) an upper limit of pH 4 during collagen extraction; (ii) a manageable viscosity of the collagen extract solution; and (iii) as few undissolved pieces of skin as possible after homogenization. Moreover, the quality objective was preservation of the native triple-helical structure of collagen.

## Figures and Tables

**Table 1:** pH values of collagen extracts at different stages of extraction for the combinations of five extraction times and four mass to volume ratios.

Extraction Time (h)	Skin: HCl (*m*/*v*) Ratio	HCl pH (Initial)	pH by the End of Extraction Time	pH by the End of Homogenization	pH Before Starting the Fibrillogenesis
					
0.5	1:10	2.04	3.24	3.99	4.20
	1:15	2.04	3.21	3.30	3.46
	1:20	2.04	3.02	2.95	3.06
	1:25	2.04	2.83	2.70	2.71
1	1:10	2.04	4.12	4.57	4.60
	1:15	2.04	3.57	3.87	3.91
	1:20	2.04	3.29	3.15	3.29
	1:25	2.05	2.92	2.83	2.83
2	1:10	1.99	3.95	4.02	4.23
	1:15	2.00	3.42	3.56	3.60
	1:20	2.00	3.34	3.42	3.48
	1:25	2.00	3.04	2.97	3.00
5	1:10	2.04	4.51	4.58	4.61
	1:15	2.00	3.92	4.11	4.21
	1:20	2.01	3.66	3.75	3.82
	1:25	2.01	3.07	3.18	3.22
8	1:10	2.00	4.82	4.90	4.93
	1:15	2.02	4.14	4.17	4.29
	1:20	2.01	3.64	3.77	3.77
	1:25	1.99	3.16	3.25	3.28

**Table 2:** ATR-FTIR peak locations of freeze-dried collagens extracted by the combinations of five extraction times and four mass to volume ratios. The means (±SD) of five replicates are given.

Amide Peak	General Range of Wavenumber (cm^−1^) *	*m*/*v* Ratio	Extraction Time				
			0.5 h	1 h	2 h	5 h	8 h
Amide A	3400–3440	1: 10	3303 (±5)	3300 (±5)	3298 (±11)	3298 (±9)	3289 (±4)
		1: 15	3298 (±5)	3298 (±6)	3299 (±4)	3301 (±3)	3294 (±7)
		1: 20	3303 (±4)	3296 (±4)	3297 (±10)	3301 (±7)	3298 (±5)
		1: 25	3298 (±6)	3300 (±3)	3298 (±8)	3297 (±5)	3293 (±10)
Amide B	near 2920	1: 10	2924 (±1)	2923 (±1)	2924 (±1)	2924 (±1)	2924 (±1)
		1: 15	2924 (±1)	2923 (±1)	2924 (±1)	2924 (±1)	2923 (±0)
		1: 20	2924 (±1)	2923 (±1)	2924 (±2)	2924 (±0)	2924 (±0)
		1: 25	2923 (±1)	2924 (±1)	2924 (±0)	2924 (±1)	2923 (±1)
Amide I	1600–1700	1: 10	1649 (±5)	1641 (±6)	1646 (±9)	1644 (±7)	1651 (±3)
		1: 15	1645 (±5)	1644 (±9)	1642 (±6)	1647 (±6)	1639 (±4)
		1: 20	1647 (±8)	1636 (±3)	1642 (±7)	1647 (±7)	1641 (±5)
		1: 25	1650 (±2)	1649 (±3)	1649 (±2)	1649 (±2)	1645 (±5)
Amide II	1550–1600	1: 10	1541 (±4)	1540 (±5)	1545 (±6)	1541 (±7)	1538 (±1)
		1: 15	1542 (±5)	1542 (±6)	1540 (±3)	1546 (±5)	1538 (±2)
		1: 20	1543 (±3)	1540 (±3)	1540 (±5)	1547 (±3)	1536 (±3)
		1: 25	1542 (±3)	1540 (±3)	1542 (±5)	1548 (±2)	1536 (±4)
Amide III	1230–1300	1: 10	1236 (±1)	1235 (±2)	1237 (±1)	1236 (±2)	1235 (±1)
		1: 15	1236 (±1)	1235 (±2)	1236 (±1)	1238 (±1)	1234 (±1)
		1: 20	1237 (±1)	1235 (±2)	1236 (±1)	1238 (±1)	1233 (±1)
		1: 25	1236 (±0)	1237 (±1)	1236 (±1)	1237 (±1)	1233 (±2)

* Sources for the general range of wavenumbers: Li et al. [6]; Wang et al. [22]; and Jeong et al. [31].

**Table 3:** Calculated absorption ratio and Rpn ratio values to confirm the triple-helical conformation, and onset temperature, denaturation temperature and enthalpy of denaturation values to assess the thermal stability of extracted collagens.

Time (h)	*m*/*v* Ratio	Absorption Ratio by FTIR	R_pn_ Ratio by CD	Onset Temperature (°C)	Denaturation Temperature (°C)	Enthalpy of Denaturation (J/g)
0.5	1:10	0.85 ± 0.01	−0.12	26.13	40.13	24.90
	1:15	0.87 ± 0.01	−0.12	22.82	41.49	21.83
	1:20	0.85 ± 0.02	−0.12	25.78	37.41	52.52
	1:25	0.86 ± 0.02	−0.12	21.46	39.44	36.07
1	1:10	0.86 ± 0.02	−0.13	21.04	41.43	40.50
	1:15	0.87 ± 0.01	−0.13	20.89	42.34	26.51
	1:20	0.86 ± 0.02	−0.13	25.89	36.03	49.15
	1:25	0.85 ± 0.01	−0.12	21.34	36.79	46.66
2	1:10	0.86 ± 0.02	−0.12	20.85	34.23	78.45
	1:15	0.86 ± 0.02	−0.12	21.76	34.66	44.59
	1:20	0.84 ± 0.01	−0.13	27.00	34.50	49.09
	1:25	0.88 ± 0.03	−0.13	21.30	34.98	40.90
5	1:10	0.85 ± 0.02	−0.12	25.82	42.12	24.06
	1:15	0.86 ± 0.01	−0.12	28.14	35.20	42.89
	1:20	0.86 ± 0.01	−0.12	26.15	35.05	54.01
	1:25	0.87 ± 0.01	−0.13	27.34	33.98	33.22
8	1:10	0.82 ± 0.02	−0.11	26.34	42.59	69.36
	1:15	0.84 ± 0.03	−0.13	30.55	42.85	36.40
	1:20	0.84 ± 0.01	−0.13	27.51	33.59	34.15
	1:25	0.85 ± 0.02	−0.13	28.35	37.70	39.79

## Data Availability

The original contributions presented in this study are included in the article. Further inquiries can be directed to the corresponding authors.

## Abbreviations

The following abbreviations are used in this manuscript:

ATR-FTIR	Attenuated Total Reflectance-Fourier Transform Infra-Red
SEM	Scanning Electron Spectroscopy
CD	Circular Dichroism
SD	Standard Deviation
DSC	Differential Scanning Calorimetry
